# Cognitive fatigability assessment test (cFAST): Development of a new instrument to assess cognitive fatigability and pilot study on its association to perceived fatigue in multiple sclerosis

**DOI:** 10.1177/20552076221117740

**Published:** 2022-08-25

**Authors:** Liliana Barrios, Rok Amon, Pietro Oldrati, Marc Hilty, Christian Holz, Andreas Lutterotti

**Affiliations:** 1 Department of Computer Science, 27219 ETH Zurich, Zurich, Switzerland; 2 27243 University Hospital of Zurich, Zurich, Switzerland; 3 27217 University of Zurich, Zurich, Switzerland; 4 542421 Neurozentrum Bellevue and Department of Neurology Hirslanden, Zurich, Switzerland

**Keywords:** Fatigue, fatigability, cognitive, mHealth, multiple sclerosis, clinical assessment, neurology, digital health, remote monitoring

## Abstract

**Background:**

Fatigue is a common symptom of many diseases, including multiple sclerosis. It manifests as a cognitive or physical condition. Fatigue is poorly understood, and effective therapies are missing. Furthermore, there is a lack of methods to measure fatigue objectively. Fatigability, the measurable decline in performance during a task, has been suggested as a complementary method to quantify fatigue.

**Objective:**

To develop a new and objective measurement of cognitive fatigability and investigate its association with perceived fatigue.

**Methods:**

We introduced the cognitive fatigability assessment test (cFAST), a novel smartphone-based test to quantify cognitive fatigability. Forty-two people with multiple sclerosis (23 fatigued and 19 non-fatigued, defined by the Fatigue Scale for Motor and Cognitive Functions) took part in our validation study. Patients completed cFAST twice. We used t-tests, Monte Carlo sampling, and area under the receiver operating characteristic curves to evaluate our approach using two sets of proposed metrics.

**Results:**

When classifying fatigue, our fatigability metric *Δresponse time* has a mean area under the receiver operating characteristic curve of 0.74 (95% CI 0.64–0.84), making it the best performing metric for this task. Furthermore, *Δresponse time* shows a statistically significant difference between the fatigued and non-fatigued groups (t = 2.27, *P* = .03). Particularly, cognitively-fatigued patients decline in performance, while non-fatigued patients do not.

**Conclusions:**

We introduce cFAST, a new instrument to quantify cognitive fatigability. Our pilot study provides evidence that cognitive fatigability assessment test produces a quantifiable drop in cognitive performance in a short period. Furthermore, our results indicate that cFAST may have the potential to serve as a surrogate for subjective cognitive fatigue. cFAST is significantly shorter than the existing fatigability assessments and does not require specialized equipment. Thus, it could enable frequent and remote monitoring, which could substantially aid clinicians in better understanding and treating fatigue.

## Introduction

### Background

Fatigue is a highly prevalent and devastating symptom of many diseases, including Parkinson's disease,^1^ multiple sclerosis (MS),^2^ and more recently, post-COVID syndrome.^3^ In MS, fatigue is rated as the most frequent and debilitating symptom.^2,4,5^ Fatigue has been defined as the subjective feeling of overwhelming exhaustion and tiredness and can manifest as a physical and cognitive symptom.^6^ The symptom is still poorly understood, and its severity can only be assessed subjectively. Currently, this is done using questionnaires such as the Fatigue Severity Scale (FSS),^7^ Modified Fatigue Impact Scale (MFIS),^8^ and Fatigue Scale for Motor and Cognitive Functions (FSMC).^9^ More than a dozen fatigue questionnaires are available.^10^ These questionnaires are used as patient-reported outcome measures in clinical trials. Their heterogeneity and subjective nature are a challenge for using them as outcome measures in clinical trials and comparing the efficacy of results across different studies. Results from different randomized placebo-controlled clinical trials testing different compounds for treating fatigue showed contradictory results, with some showing good efficacy, and others exhibiting no effect.^11–18^

### Fatigue and fatigability

The perception of fatigue (subjective measurement) is being differentiated from performance fatigability (objective measurement).^19^ Fatigability is further divided into the motor and cognitive domains. Motor fatigability has been quantified as the decline in peak performance, power, or speed during physical activity.^20^ On the other hand, cognitive fatigability measures the decline of cognitive performance during a task that requires sustained attention,^20^ and it has been measured as an increase in reaction time, decline in accuracy or by comparing the performance during the first and last third of a task.^21,22^ Establishing an association between objective fatigability and subjective fatigue is an important goal for clinical research but has been proven difficult.^19^ While a correlation between motor fatigability and perceived fatigue has been suggested in several studies,^23–27^ less data is available on cognitive fatigability.^28–35^ A possible cause is the complexity of inducing cognitive fatigability and the lack of consensus and dedicated tests to quantify it.^22^ Prior studies used one of two strategies to generate cognitive fatigability. Either they conducted a test battery, including the same test before and after fatiguing tasks and compared their performance, or they employed a single prolonged cognitive task and measured the decline in performance within the task. Some of the used cognitive tests within fatigability research include: (1) the Paced Auditory Serial Addition Test (PASAT),^36^ (2) the Psychomotor vigilance task (PVT),^37^ and (3) the Stroop test.^38^ However, utilizing these non-specific cognitive performance tests to assess cognitive fatigability comes with certain drawbacks, such as long testing sessions.

### Limitations of cognitive fatigability studies

Fatigability in healthy subjects is typically studied through long examination sessions. Van der Linden et al. induced fatigue through two hours of cognitively demanding tasks. Their study showed a significant difference in planning ability and increased perseverative errors between the non-fatigued and fatigued participants.^32^ Other cognitive fatigability studies in healthy subjects using the Stroop test employed a study length of 3 and 2 h for young adults^34^ and for older adults,^35^ respectively. However, long testing sessions are not unique to healthy subjects. Moeller et al. administered two hourly test batteries for analyzing cognitive fatigability using three neuropsychological tests in subjects with mild traumatic brain injury.^33^ In MS, there is large heterogeneity when it comes to studying cognitive fatigability. DeLuca et al.^39^ studied fatigue in 15 people with MS (pwMS) and 15 controls by conducting four modified Symbol Digit Modality Test (mSDMT) trials over an hour of fMRI scanning where users were shown different symbol–digit pair probes at varying interstimulus. Participants had to respond “match” or “no match” to each probe by following a provided symbol-digit arrangement. The interstimulus interval randomly varied between 0, 4, 8, and 12 s. Results from their study found no cognitive fatigability. Chen et al.^40^ also studied fatigability using an mSDMT within a fMRI setting. During examination pwMS and controls completed a total of eight mSDMT (four with high cognitive load and four with low cognitive load), each lasting 7.7 min. The authors did not study within trial performance, but across trial performance showed an increase in reaction time associated with subjective fatigue in pwMS. Berard et al. compared the performance during quintiles of a 20 min PVT session to quantify cognitive fatigability and found a greater increase in reaction time of patients compared to healthy controls.^31^ PVT is a simple reaction time task where participants have to press a button in response to the presence of a stimulus. However, its repetitive and monotonous nature often results in participants reporting feelings of boredom,^41^ and thus the performance decline may be influenced by a lack of motivation rather than fatigability.^42^ Finally, several authors employed the PASAT by comparing the decrease in accuracy between the beginning and end of the test.^28–30,42–44^ Even though the PASAT is applied in many studies, there is still significant methodological heterogeneity. First, some studies compared the performance between the first and the second half of the test^28^, while others compared the performance between thirds.^29^ Second, despite there seems to be a general consensus of 3 s length inter-stimulus interval (ISI), this has not been uniformly applied in fatigability studies.^20,30,44^ Third, it is known that pwMS may adopt a “chunking strategy,” particularly as task demands increase,^45^ meaning that they add two numbers, skip one, and add the following two, thus, reducing the overall difficulty of the task by decreasing the simultaneous cognitive load. Only recently, first normative data on cognitive fatigability has been generated to account for the chunking strategy.^43^ Fourth, the PASAT requires a medical examiner to conduct the test, making it more expensive to administer. Finally, patients have described the PASAT as unpleasant and causing anxiety,^46^ limiting the applicability and repeatability of the tests.

### Aims and overview of the study

We propose a new test for measuring cognitive fatigability in a short period (i.e. 5 min) and refer to it as the Cognitive Fatigability Assessment Test (cFAST). cFAST is inspired by the Symbol Digit Modality Test (SDMT) digit-symbol matching logic.^47^ SDMT is a cognitive test that measures information processing speed. Studies showed that the SDMT is relatively resistant to practice effects,^48^ in particular when rearranging the keys,^49^ making it an attractive tool for cognitive monitoring over time in clinical trials.^50^ Moreover, it has also been validated for smartphones.^51^ Our study uses a similar key-symbol matching strategy to measure fatigability instead of cognitive impairment.

The goals of this feasibility study were two-fold. The first goal was to develop an objective and ubiquitous measurement of cognitive fatigability. We achieved this goal by implementing a smartphone-based test through an iterative process involving patients, neuropsychologists and neurologists. We opted for a smartphone-based implementation given the high acceptability and interest of pwMS in smartphone-based tools that allow them to monitor and manage their condition.^52–59^ The second goal was to study the association between the newly developed objective measurement (cFAST) and perceived cognitive fatigue. We approached this goal by conducting a pilot study with pwMS who completed the cFAST and the FSMC.^9^ Using the FSMC cognitive subscale, we assign the participants to the cognitive-fatigued (subscale> = 22) and non-cognitive-fatigued (subscale<22) groups.^9^ From the cFAST, we extracted a set of metrics and evaluated group differences with t-tests. Through area under the receiver operating characteristics (AUROC), we assessed the performance of our proposed metrics to classify cognitive fatigued versus non-cognitive-fatigued patients. Furthermore, we investigated the relationship of our proposed test (cFAST) and metrics to disability.

## Methods

### Development of the smartphone-based test, cFAST

We aimed to develop a test to objectively quantify cognitive fatigability, that meets the requirements: (1) engages cognitive processing speed and induces cognitive load, (2) is short, self-explanatory, and allows for remote monitoring, and (3) does not require medical supervision. We followed an iterative process during the design and development of the application. The medical professionals reviewed different prototypes to ensure an appropriate design based on clinical theory and practice is implemented. Additionally, we gathered informal feedback from people with MS (pwMS) regarding our prototypes before converging on our final design. Refer to the Supplements for further details on the prototypes designs and selection.

**Figure 1.**
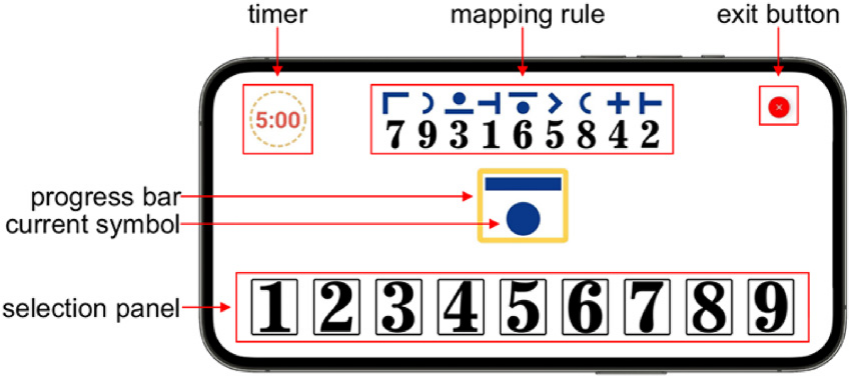
cFAST user-interface with highlighted elements in red. *Note.* cFAST, cognitive fatigability assessment test.

 Figure 1 displays the user interface of the cFAST and highlights each of its elements. The test is designed to be carried out by holding the smartphone in landscape mode. The middle of the screen shows a large blue symbol (main symbol). The main symbol has to be mapped to its corresponding digit following the mapping rule displayed at the top of the screen. Selection occurs by tapping the numbers located at the bottom of the screen. Users have a limited time to find the corresponding number associated with the main symbol. A yellow progress bar around the symbol indicates how much time is left until the symbol is changed automatically. The main symbol changes under two circumstances: (1) after the user taps a number or (2) when the progress bar has entirely run out. Every time a new symbol appears, the associations and positions of the top mapping rule are randomized, and the progress bar is restarted. The randomization seeks to diminish the possibility of a learning effect associated with memorizing the digit-symbol mapping within the same test run. The progress bar works as a pressure mechanism to motivate users to be fast and avoid resting periods. A timer located at the top left indicates how much time is left for the test to end. Users can exit the test at any moment by tapping the exit button located at the top right corner. If exited early, the test is considered invalid.

Our test is inspired by the SDMT,^47^ as it is a widely used, accepted, and validated cognitive assessment test in MS. However, cFAST differs from the SDMT in several aspects:

1. cFAST is a cognitive fatigability test, while SDMT assesses cognitive impairment and working memory.
2. Contrary to the SDMT, cFAST does not allow participants to look ahead to match the following symbols. Hence, participants have no way to anticipate the next answer to reduce their response time.
3. There is a time limit to complete each selection in cFAST.
4. cFAST randomizes the matching rules after each answer, while SDMT has a fixed matching rule.
5. The duration of a cFAST session is 5 min, while the SDMT lasts 90 s. The increased duration is needed because cognitive fatigability is notoriously hard to elicit in a short time. However, cFAST is comparatively significantly shorter than previous attempts at measuring cognitive fatigability.

All these design considerations seek to evaluate cognitive fatigability.

### Application logic

**Figure 2.**
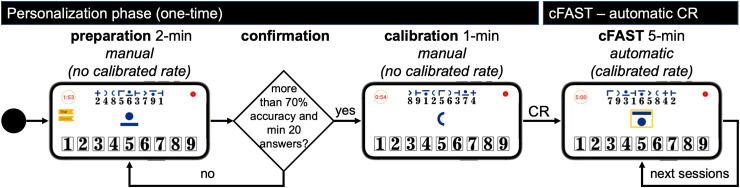
cFAST application logic. In the personalization phase, users complete the preparation and confirmation to ensure they understand the test's matching logic and the calibration to derive the *calibrated rate* used in cFAST. After this phase, cFAST is personalized and ready to be used. *Note.* cFAST, cognitive fatigability assessment test; CR, calibrated rate.

cFAST is designed with the aim of being conducted outside the clinic and without medical supervision. Therefore, the application logic is self-explanatory and contains a personalization phase to maximize the users’ understanding and tailor it to their performance. This phase needs to be completed before being able to run cFAST. Figure 2 depicts the application logic diagram.

At the start of the personalization phase, users are prompted for a mandatory two-minute preparation step. The goal of this step is for users to familiarize with the test matching logic and rules before starting the calibration step. To this end, a confirmation step ensures that, during the preparation, users provided at least 70% correct digit-symbol matches out of a minimum of 20 answers. Contrary to the calibrated cFAST, there is no time limit to match individual symbols during preparation. Hence, symbols only change after the user presses a number from the selection panel. We refer to this method as *manual*. This functionality allows users to understand the test matching logic without time pressure.

**Figure 3.**
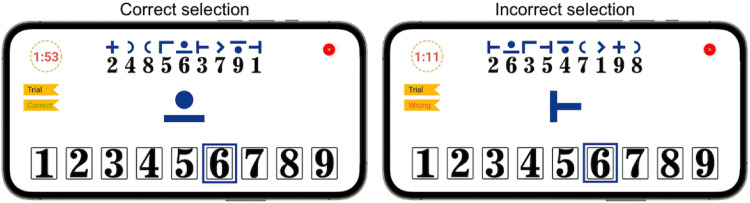
Preparation step user interface. The blue rectangle indicates the answer provided by the user. After each number selection, the interface indicates with a label whether their attempt is **correct** (left) or **wrong** (right).

During the preparation, users receive immediate feedback on whether their choice is correct or incorrect through a label located at the left side of the screen (cf. Figure 3). Failed preparation trials indicate that the user has not sufficiently trained in operating the test yet or did not perform it as fast as possible and thus must repeat it. The motivation for providing immediate feedback is to help the user understand the matching mechanics of the test. This functionality is particularly beneficial for unsupervised settings where no medical examiner is present to clarify doubts to the patients. Users can start the calibration step only after preparation is passed successfully. The calibration step lasts one minute and it uses the same logic of the preparation step, but without providing feedback. At this point, we assume users understand the test matching logic. Similar to preparation, calibration also employs a manual mechanism. However, its goal is to extract the users’ reaction time, which we call *calibrated rate.* This rate is then used in cFAST. Thus, the manual function of the application has two goals: (1) during the preparation it allows sufficient time for users to understand the test matching logic, and (2) during calibration, it helps derive a personalized *calibrated rate*.

#### Deriving calibrated rate

The calibrated rate is a key feature of cFAST and it is derived from the 1-min calibration step of the personalization phase (Figure 2). The calibration step has the same logic of the preparation but without user feedback. During calibration, symbols are only changed once the user taps a number from the selection panel (manual mechanism). We use 85% percentile of the response time exhibited during the calibration step to extract the calibrated rate, meaning each individual user may perform the task at different rates but always in relation to their top performance. Thus, the calibrated rate is tailored to each user, accounting for patients’ different levels of disability. Once the calibrated rate is derived, cFAST is personalized and ready to use.

#### Eliciting cognitive fatigability

**Figure 4.**
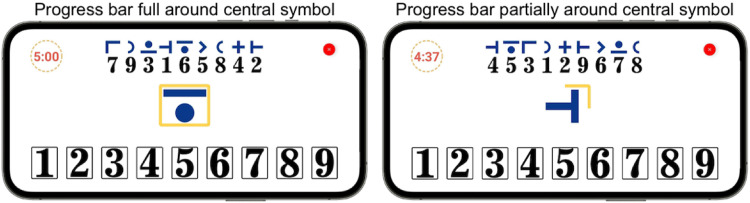
cFAST user interface. The left side of the image displays the screen at the beginning of a 5-min test The yellow progress bar indicates the remaining time to complete a selection. The digit-symbol mapping is randomized after each selection to reduce learning effects. *Note.* cFAST, cognitive fatigability assessment test.

During a cFAST session, users are supposed to repeatedly match a symbol with their corresponding number. However, tasks of this nature are typical examples of speed-accuracy trade-off.^60^ Participants tend to decide between performing the test with high accuracy but slow (i.e. low exertion) or fast but with low accuracy. Either of these scenarios would significantly limit the fatigue-inducing effect of the test. With cFAST, we seek to reduce this trade-off by adding a limited timeframe (calibrated rate) for each selection. This timeframe is indicated through a yellow progress bar (Figure 4). With this approach, participants cannot spend unlimited time making a decision. Moreover, we hypothesize that the added pressure to make a fast selection contributes to the cognitive load required to induce cognitive fatigability.

### Participants

We recruited 48 patients from the MS outpatient clinic of the Department of Neurology, University Hospital Zurich, between September 2020 and April 2021. Participants provided written consent following the Declaration of Helsinki.^61^ The expanded disability status scale (EDSS) was obtained from the routine neurologic examinations performed at the hospital. This study was approved by the local ethics committee (Cantonal Ethics Committee Zurich, Switzerland). Inclusion criteria consisted of: (a) confirmed MS diagnosis and (b) age between 18 to 70. In addition, exclusion criteria included: diagnosis of depression, schizophrenia, bipolar disorders, attention deficit hyperactivity disorder, and regular intake of psychostimulants or anticonvulsant medications.

### Procedure

Participants were briefly introduced to the study setup and completed a demographic questionnaire. Following, the study examiner showed them the application and the logic of the cFAST. Participants started with the 2-min preparation session. After successful completion, they performed the calibration step. Next, we asked participants to complete a first cFAST session of 5 min that is considered as a trial, to ensure they understand the test logic. Following, there was a short break in which participants filled out the FSMC questionnaire. Next, participants performed a second cFAST session. Previous cognitive fatigability studies including modified versions of the SDMT do full trials and discard this data before conducting the actual test to ensure participants understand the test logic.^40^ Hence, all data analyses presented in this paper are based on the main cFAST and not on the trial data.

### Data collection and processing pipeline

We collected touch data from the smartphone using a custom Android application that we developed. Each sample in our dataset contains the ID of the symbol to be matched, the user's selection if there was any, the current mapping rule, and the timestamp of the touch-down event. Our data processing pipeline includes three steps: (1) artifact detection, (2) cognitive adaptation removal, and (3) metrics extraction.

1. Artifact detection
  We use *response time* as one of our primary performance metrics. Artifacts in *response time* typically appear when a user aims at tapping a digit to match the current symbol, but they run out of time. Hence, the newly displayed symbol is stored with a short *response time*, and the previous symbol is marked as a *missed answer* (Figure 5 left). These artifacts need to be identified and removed to avoid double-counting errors and compute a misleading *response time*. Therefore, in our preprocessing step, we remove any entry after a missed answer with a *response time* of less than the average minus two standard deviations of the entire cFAST session's *response time*. This results in subject-specific thresholds that account for the difference in average performance. With this method, we remove an average of 3.8 entries per session, with the average session containing 138 answers. Figure 5 right shows the same data after artifact removal.
2. Cognitive adaptation removal.
  Previous cognitive fatigue studies describe the existence of an adaptation phase occurring at the beginning of a cognitive task due to some unspecific modulations of training and adaptation and highlight the need to account for these effects when studying fatigue.^62,63^ A common strategy to deal with the adaptation in cognitive fatigue studies is to omit the start of the task.^62,63^ An adaptation phase is not unique to cognitive tasks as it has also been detected in motor fatigability tasks. A similar strategy is applied in motor tasks by removing the start of the task to account for the adaptation period.^27,64,65^ cFAST sessions exhibit an adaptation period in the initial part of the test, in particular for fatigued patients. Figure 6 depicts the average mean-normalized response times for all fatigued pwMS for the whole 5 min cFAST in 30 s segments. During the first segments, we observe an increase in response time, followed by a decrease in response time in the third segment. We attribute these changes in performance to an adaptation period before users are fully immersed in the test.^62,63^ Hence, to make a fair comparison between the study participants we discard the first 60 s of all cFAST tests (42 sessions) before extracting the metrics and performing the data analysis.

**Figure 5.**
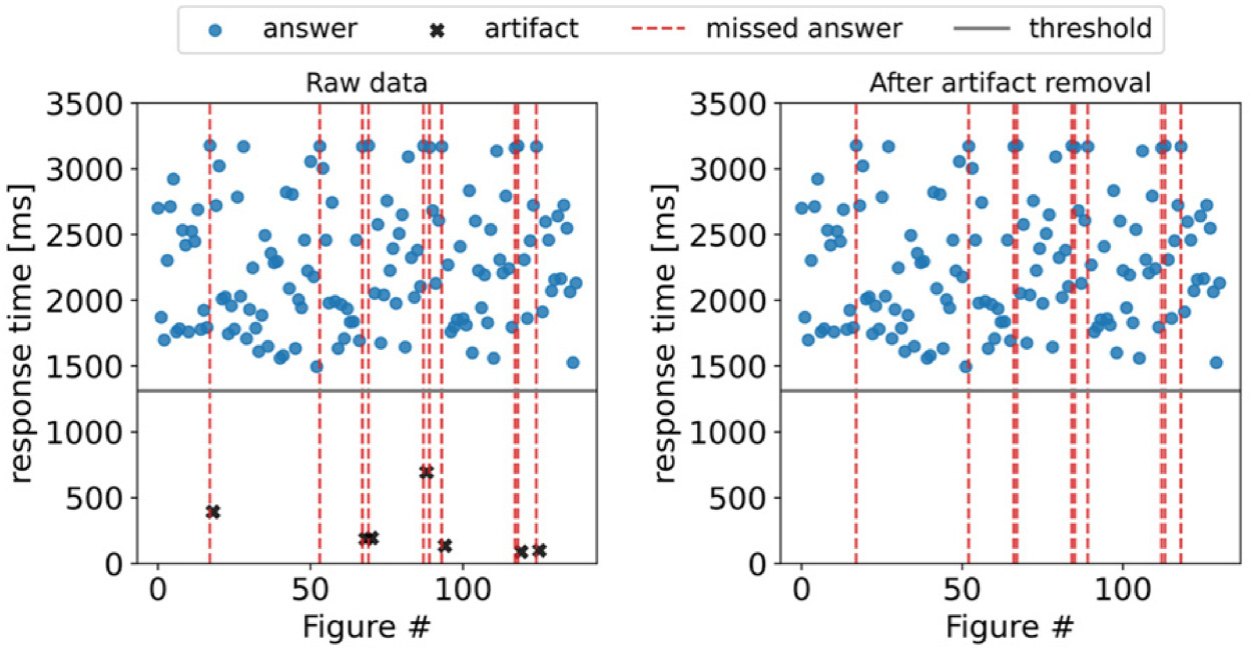
Artifacts in *response time* typically appear when a user provides an answer shortly after running out of time. Therefore, the pressed digit is associated with the newly displayed figure. As a result, the previous entry is classified as a missed answer, and the current figure has a very short *response time* (left side). We detect and remove these artifacts to avoid misleading *errors* and *response time* values (right side).
3. Metrics extraction
  **Figure 6.**
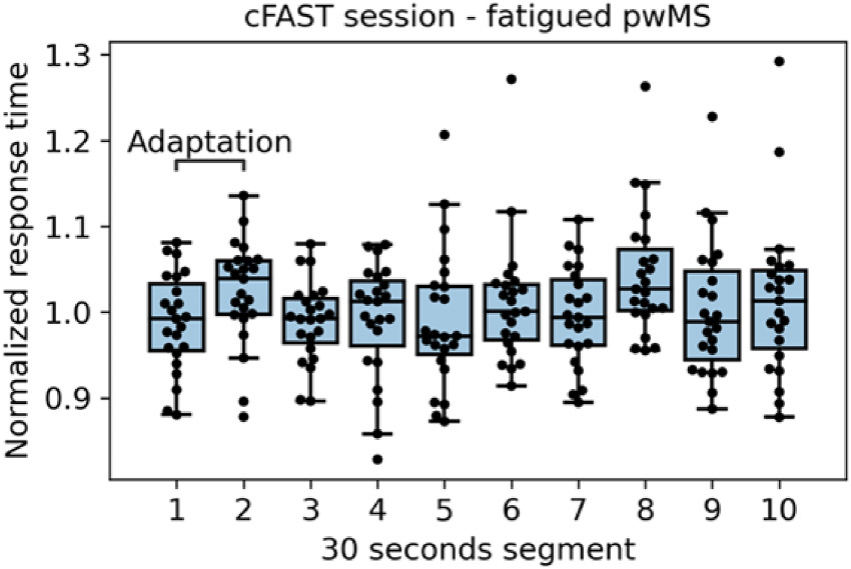
cFAST session with average mean-normalized reaction time per 30 s segments for each fatigued participant. The first two segments (60 s) are discarded as we consider them part of the adaptation phase. *Note.* cFAST, cognitive fatigability assessment test.

We define two sets of metrics to quantify performance during a cFAST test session: (1) *general* metrics, which represent the average performance during an entire test session, and (2) *fatigability* metrics, which measure the change in performance occurring between the first third and last third of a test session. Table 1 displays an overview of the proposed metrics with their definition.

**Table 1.** Metrics description.

Metric name		Description
**General**		
	*Response time*	Average time in milliseconds to tap a digit from the selection panel after the appearance of a new symbol
	*Calibrated rate*	Time duration in milliseconds for each new symbol – derived from the calibration phase (corresponds to progress bar duration)
	*Correct*	Total correct matches
	*Errors*	Total errors including wrong matches and missed answer
**Fatigability**		
	*Δcorrect*	Percent change in *correct* between the first and the last third of the task.
	*Δresponse time*	Percent change in *response time* between the first and the last third of the task.
	*Δ*errors	Percent change in *errors* between the first and the last third of the task.

### Statistical analyses

We use descriptive statistics to summarize and compare the study subpopulations. We evaluate the performance of our derived smartphone-based metrics to discriminate between cognitive-fatigued and non-cognitive fatigued subjects following the FSMC cognitive subscale (threshold = 22).^9^ With t-tests, we explore group differences and consider *P* < .05 significant. Furthermore, through AUROC, we evaluate the performance of our derived smartphone-based metrics to classify cognitive fatigued versus non-cognitive fatigued subjects, independently of age and EDSS. We assess the robustness of our approach and compute confidence intervals for AUROC using stratified Monte-Carlo sampling^66^ with 1000 iterations and randomly select (without replacement) in each iteration 1/2 of our participants’ data (cFAST sessions) for evaluation. We partition the cFAST data into eight strata, following two partitioning criteria: (a) cognitive fatigued as a binary state according to FSMC cognitive subscale (threshold = 22) and (b) an EDSS group, which can be one of four: [0,1), [1, 2), [2, 3), and [3,∞). The idea of this partition is to find a metric that works best in the whole spectrum of disability. Each participant and their data are fully assigned to one of the resulting eight strata. Thus, when performing the stratified split, either a participant's data is fully contained in the split or not at all. Hence, with our approach, we split at the participant level, ensure class balance, and account for disability. Additionally, as age also influences cognitive performance,^33^ we create eight additional strata following two partitioning criteria: (a) cognitive fatigued as a binary state and (b) age group, which can be one of four: (18, 30), [30,40), [40,50) and [50, 70]. This partition aims at reducing the influence of age in the metrics by assigning weights according to the group sizes. Furthermore, we use one-way analysis of covariance (ANCOVA) with EDSS as a covariant to rule out the effect of disability when analyzing fatigue.

Finally, we further explore how cFAST and our proposed metrics relate to disability by measuring the performance of the metrics to rate disability according to EDSS. To this end, we split the study participants in two groups according to EDSS and analyzed the difference in performance between both groups. We classify patients with EDSS>1.5 as disabled and patients with EDSS< = 1.5 not disabled. For this evaluation, we partition our dataset into four strata, following two partitioning criteria: (a) disabled as a binary state according to the EDSS (0 for EDSS< = 1.5 and 1 for EDSS>1.5), and (b) cognitive fatigued as a binary state according to FSMC cognitive subscale (threshold = 22). Additionally, we use the same age groups as we did for the cognitive fatigue evaluation. We report the average AUROC with 95% confidence intervals. In addition, we include plots of the ROC curves for visual inspection.

## Results

### Participant characteristics

**Figure 7.**
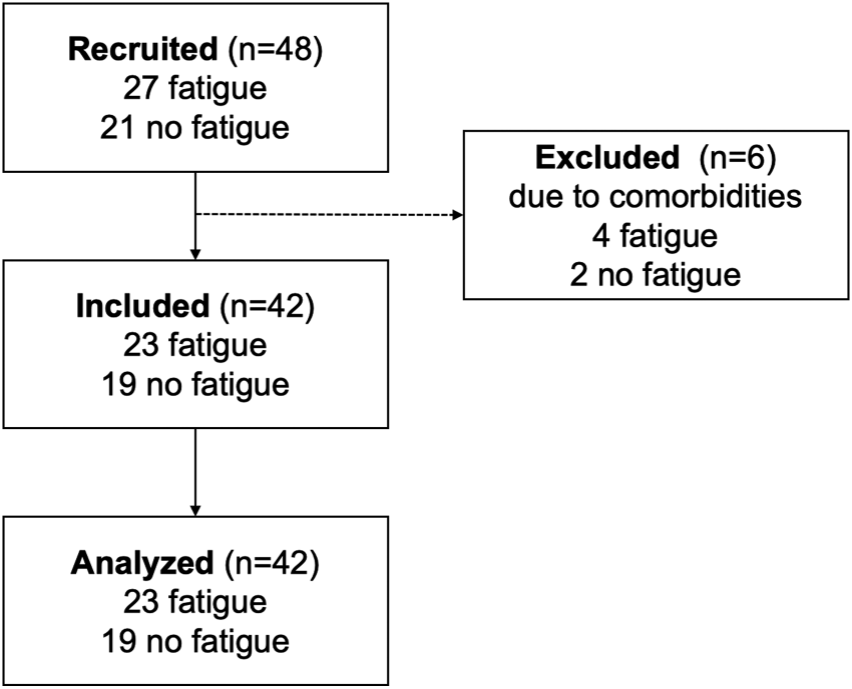
Flow chart of the study and overview of excluded participants.

We recruited 48 study participants and from those we excluded 6 due to comorbidities including iron deficiency, personality disorder, hypothyroidism, and narcolepsy type 1. Table 2 summarizes the study participants divided into the two subgroups of interest (i.e. no cognitive fatigue and cognitive fatigue according to the FSMC subscore). Of the recruited pwMS, 21 did not have cognitive fatigue and 27 were cognitively fatigued. Of those we included in our analysis, 19 participants did not have fatigue and 23 were fatigued. Figure 7 shows the flow chart of the study and an overview of the excluded patients. The gender distribution of the participants in the two groups, the mean and standard deviation of their age, EDSS, and the FSMC subscales are listed in Table 2. As expected, we found a significant difference in all the FSMC scores. However, we found no statistically significant difference between the age and gender distributions of the two groups.

**Table 2.** Demographic characteristics of participants.

		No fatigue	Cognitive fatigue	*P*
Number		19	23	
Age, mean (SD)		36.89 (12.15)	38.22 (12.20)	.73
**Gender, n (%)**				
	m	8 (42)	6 (26)	.44
	w	11 (58)	17 (74)	
**MS type, n (%)**				
	PMS	1 (5)	3 (13)	.61
	RRMS	18 (95)	20 (87)	
**Disease duration, mean (SD)**		9.63 (5.88)	12.52 (8.51)	.20
**DMT, n (%)**				
	None	1 (5)	1 (4)	
	Interferon beta-1a	1 (5)	0 (0)	
	Dimethyl fumarate	2 (11)	1 (4)	
	Teriflunomide	1 (5)	1 (4)	
	Glatiramer acetate	1 (5)	1 (4)	
	Fingolimod	1 (5)	1 (4)	
	Natalizumab	6 (32)	8 (35)	
	Rituximab	1 (5)	3 (13)	
	Ocrelizumab	5 (26)	7 (31)	
**Fatigue medication, n (%)**				
	None	19 (100)	22 (96)	1.00
	Modafinil	0 (0)	1 (4)	
EDSS, mean (SD)		1.00 (1.18)	2.41 (1.95)	.006
**FSMC, mean (SD)**				
	Total	30.84 (8.00)	64.30 (16.29)	<.001
	Cognitive	14.26 (3.25)	31.70 (8.81)	<.001
	Motor	16.58 (5.60)	32.61 (8.68)	<.001

Data are mean (SD) or n (%).

*Note.* PMS, progressive multiple sclerosis; RRMS, relapsing-remitting multiple sclerosis; Disease duration is measured in years since first manifestation; EDSS, expanded disability status scale; FSMC, fatigue score for motor functions and cognition; DMT: disease-modifying therapy.

### Correlation to clinical data

Our analysis indicates a significant Spearman rank correlation between several of the proposed general metrics and the clinical data. Table 3 shows an overview of all the computed correlations. The *response time* and *correct* metrics showed the highest correlation with EDSS (ρ = 0.6, *P* < .001 and ρ = -0.6, *P* < .001, respectively). Then, *calibrated rate* follows with ρ = 0.5, *P* = .001. On the other hand*, errors* did not significantly correlate to EDSS (ρ = -0.07, *P* = .67). We also found a significant correlation when analyzing the relationship between our metrics and the FSMC cognitive subscore. Again, *response time* and *correct* showed the highest correlation to the FSMC subscore (ρ = 0.39, *P* = .01 and ρ = -0.38, *P* = .01, respectively). Neither *calibrated rate* (ρ = 0.27, *P* = .09) nor *errors* (ρ = 0.1, *P* = .51) significantly correlated to the FSMC cognitive subscore. Age also correlates to the proposed general performance metrics. Among the correlating metrics, we found *correct* (ρ = -0.66, *P* < .001), *response time* (ρ = 0.61, *P* < .001), and *calibrated rate* (ρ = 0.51, *P* = .001). We found no significant correlation between the fatigability metrics and the clinical data.

**Table 3.** Spearman rank correlation coefficient ρ: metrics vs. clinical data.

	EDSS	FSMC cognitive score	Age
*Response time*	0.6 (<.001)	0.39 (.01)	0.61 (<.001)
*Calibrated rate*	0.5 (.001)	0.27 (.09)	0.51 (.001)
*Correct*	−0.6 (<.001)	−0.38 (.01)	−0.66 (<.001)
*Errors*	−0.07 (.67)	0.1 (.51)	0.01 (.93)
*Δcorrect*	−0.03 (.86)	−0.21 (.17)	0.08 (.59)
*Δresponse time*	0.21 (.17)	0.24 (.13)	0.08 (.59)
*Δerrors*	−0.13 (.40)	0.13 (.42)	−0.2 (.19)

*Note.* Data are ρ (*P*). EDSS, expanded disability status scale; FSMC, fatigue score for motor functions and cognition.

### cFAST relationship to perceived fatigue

We investigated the relationship between our metrics and perceived fatigue by determining statistically significant differences between the cognitive-fatigued and non-fatigued groups. Table 4 depicts a complete overview of the metrics’ mean value and standard deviation for both groups, as well as the t-test results. Table S2 in the Supplements includes the non-parametric testing results using Mann Whitney U.

**Table 4.** Metrics comparison between fatigued and non-fatigued patients with mean (SD), independent samples t-test (two-tailed) to assess whether there is a statistically significant difference between the groups, and Cohen's d effect size.

	No fatigue (n = 19)	Cognitive fatigue (n = 23)	t	*P*	*Cohen's d*
Response time*	2083.3 (358.31)	2586.88 (961.28)	2.16	.04	0.669
Calibrated rate**	3289.47 (1229.75)	3922.91 (1396.06)	1.54	.13	0.478
*Correct*	109.11 (15.97)	90.96 (24.21)	−2.8	.008	−0.868
*Errors*	7.58 (6.07)	8.04 (4.13)	0.29	.77	0.091
*Δcorrect*	3.51 (11.19)	−2.73 (9.95)	−1.91	.06	−0.593
*Δresponse time*	−0.96 (5.5)	2.69 (4.94)	2.27	.03	0.703
*Δerrors*	−0.46 (2.05)	0.03 (1.86)	0.81	.42	0.252

*Response time is not normally distributed for the subgroup cognitive fatigue.

**Calibrated rate is not normally distributed for the subgroups.

We found a significant difference between both groups regarding *response time* (t = 2.16, *P* = .04, d = 0.669). The group with cognitive fatigue had an average response time of 2586.88 (SD = 961.28) ms, compared to the 2083.3 (SD = 358.31) ms of non-fatigued participants. We did not find a statistically significant difference in *calibrated rate* (t = 1.54, *P* = .13). Furthermore, we found that *correct* differed significantly between the groups (t = -2.8, *P* = .008, d = -0.868). The non-fatigued participants gave an average of 109.11 (SD = 15.97) correct answers, while the fatigued group had an average of 90.96 (SD = 24.21) correct answers. However, *errors* was not significantly different between the groups (t = 0.29, *P* = .77).

In terms of the fatigability metrics, we found that *Δresponse time* significantly differed between the groups (t = 2.27, *P* = .03, d = 0.703). On average, fatigued participants had a *Δresponse time* of 2.69 (SD = 4.94) ms, while non-fatigued participants had an average *Δresponse time* of −0.96 (SD = 5.5) ms. *Δerrors* and *Δcorrect* did not show a statistically significant difference between the groups (t = 0.81, *P* = .42 and t = -1.91, P = .06, respectively).

**Figure 8.**
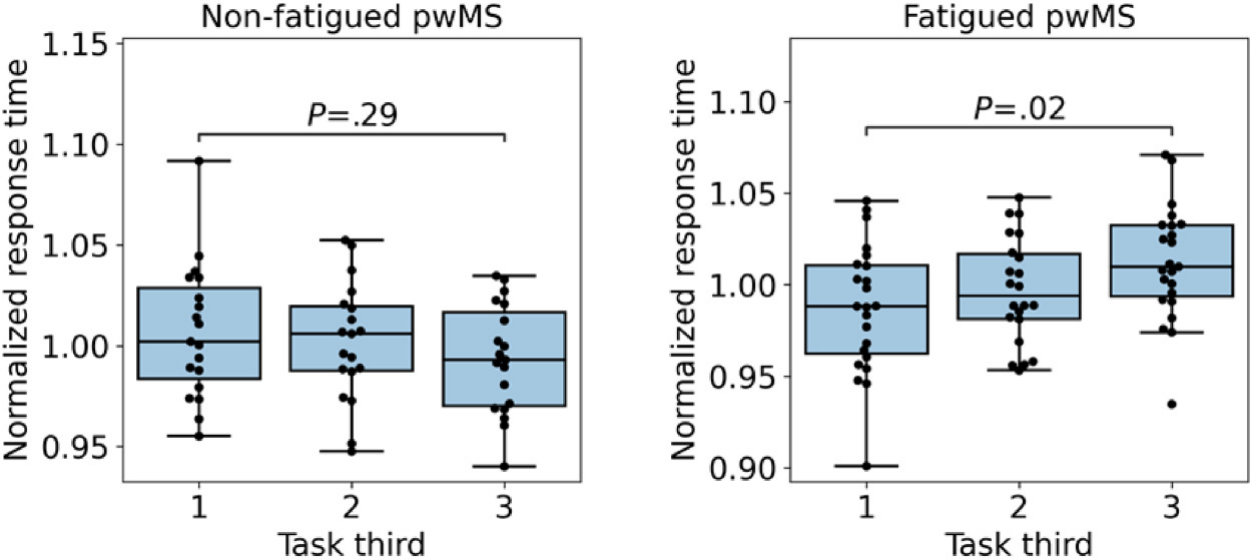
Average normalized response time during the three thirds of the cFAST session data after preprocessing for non-fatigued pwMS (left) and fatigued pwMS (right). A significant increase in the response time between the first and the last third of the task is present for fatigued patients only. The thirds were compared using a paired t-test. *Note.* cFAST, cognitive fatigability assessment test; pwMS, people with multiple sclerosis.

To analyze the temporal progression of participants’ performance during a cFAST session, we performed a series of paired t-tests. Figure 8 on the left depicts the average normalized response time in the three thirds of the session for non-fatigued pwMS. While Figure 8, on the right, shows the results for pwMS with cognitive fatigue. For the group with no fatigue, the results are primarily flat and with a slight trend to improve over time, while for the fatigued group, we see a significant increase in response time (*P* = .02) between the first and last third of the session.

### cFAST relationship to disability

The disabled group has a mean EDSS of 3.26 (SD = 1.54) and the non-disabled group has a mean EDSS of 0.54 (SD = 0.67). Detailed demographics of these groups is described in Table S1 in the supplements. Table 5 shows a complete overview of the metrics’ average value and standard deviation for both groups, as well as the t-test results. Table S3 in the Supplements includes the non-parametric testing results using Mann Whitney U.

**Table 5.** Metrics comparison between disabled and not disabled patients with mean (SD), independent samples t-test (two-tailed) to assess whether there is a statistically significant difference between the groups, and Cohen's d effect size.

	Not disabled (n = 23)	Disabled (n = 19)	t	*P*	*Cohen's d*
Response time*	2080.23 (317.39)	2696.61 (1030.37)	2.47	.02	0.844
Calibrated rate**	3211.22 (840.63)	4151.0 (1658.95)	2.38	.02	0.737
*Correct*	108.3 (15.1)	88.11 (25.44)	−3.19	.003	−0.989
*Errors*	7.96 (5.69)	7.68 (4.26)	−0.17	.86	−0.053
*Δcorrect*	0.55 (10.68)	−0.47 (11.35)	−0.3	.77	−0.093
*Δresponse time*	0.29 (5.55)	1.95 (5.33)	1.98	.33	0.304
*Δerrors*	−0.03 (2.05)	−0.4 (1.83)	−0.6	.55	−0.187

*Response time is not normally distributed for the subgroup disabled. *Levene's* test *P* < .05 equal variance not assumed. **Calibrated rate is not normally distributed for the subgroups.

We found a significant difference in *response time* between the groups (t = 2.47, *P* = .02, d = 0.844). Participants without disability had an average *response time* of 2080.23 (SD = 317.39) ms, compared to the 2696.61 (SD = 1030.37) ms exhibited by the disabled pwMS. Similarly, *calibrated rate* was significantly lower for participants without disability (t = 2.38, *P* = .02, d = 0.737), with an average of 3211.22 (SD = 840.63) ms against the 4151.0 (SD = 1658.95) ms of disabled participants. Consequently, *correct* followed the same trend (t = -3.19, *P* = .003, d = -0.989). On average, disabled pwMS provided 88.11 (SD = 25.44) correct answers, compared to the higher 108.3 (SD = 15.1) of participants without disability. We found no significant difference in *errors* (t = -0.17, *P* = .86).

We performed the same analysis with the fatigability metrics. *Δcorrect*, *Δresponse time*, and *Δerrors* showed no statistically significant difference between the not disabled and disabled groups (respectively t = -0.3, *P* = .77, t = 1.98, *P* = .33 and t = -0.6, *P* = .55).

### Predictive power of the cFAST metrics to classify cognitive fatigue

To further explore the association between cognitive fatigability and perceived fatigue, we assessed the predictive power of our metrics to classify cognitive fatigue participants according to the FSMC cognitive subscale. Table 6 shows the results corresponding to the mean AUROC with its respective confidence intervals. The results indicate that the best features for fatigue independently of the EDSS are the fatigability metrics. *Δresponse time* had the highest AUROC with 0.74 (95% CI 0.64–0.84). Following, *Δcorrect* and *Δerrors* with an average AUROC of 0.72 (95% CI 0.63–0.85) and 0.65 (95% CI 0.53–0.77), respectively. From the general metrics, *response time* performed the best with a mean AUROC of 0.63 (95% CI 0.50–0.76). The *correct* metric had an AUROC of 0.62 (95% CI 0.50–0.74). *Calibrated rate* produced an AUROC of 0.59 (95% CI 0.44–0.74). The *errors* metric showed an AUROC of 0.58 (95% CI 0.44–0.72). Age had an average AUROC of 0.58 (95% CI 0.47–0.69). Lastly, EDSS had an AUROC of 0.53 (95% CI 0.43–0.63).

**Table 6.** AUROC score corresponding for cognitive fatigue classification according to the FSMC cognitive subscale for the proposed metrics (sorted by AUROC in descending order).

	Metric name	↓AUROC (95% CI)
Fatigability	*Δresponse time*	0.74 (95% CI 0.64-0.84)
Fatigability	*Δcorrect*	0.72 (95% CI 0.63-0.85)
Fatigability	*Δerrors*	0.65 (95% CI 0.53-0.77)
General	*Response time*	0.63 (95% CI 0.50-0.76)
General	Correct	0.62 (95% CI 0.50-0.74)
General	*Calibrated rate*	0.59 (95% CI 0.44-0.74)
General	*Errors*	0.58 (95% CI 0.44-0.72)
Demographic	*Age*	0.58 (95% CI 0.47-0.69)
Demographic	EDSS	0.53 (95% CI 0.43-0.63)

### Predictive power of the cFAST metrics to classify disability

To evaluate the best cFAST metrics to classify disability independently of fatigue, we performed the same analysis as we did for cognitive fatigue. Results suggest that the general metrics are better than the fatigability metrics for disability in terms of AUROC. A complete overview of these results is shown in Table 7. *Response time* produced an average AUROC of 0.64 (95% CI 0.50–0.78), followed by *age* with an average AUROC of 0.63 (95% CI 0.53–0.73). Following, *correct* showed an average AUROC of 0.63 (95% CI 0.49–0.77). *Calibrated rate* had an average AUROC of 0.59 (95% CI 0.43–0.75). *Δerrors* had a mean AUROC of 0.55 (95% CI 0.41–0.69). Following, *Δcorrect* produced an AUROC of 0.52 (95% CI 0.38–0.66). AUROC of *errors* for disabled patients was 0.51 (95% CI 0.38–0.64). Finally, *Δresponse time* was the worst metric for disability with an average AUROC of 0.50 (95% CI 0.36–0.64).

**Table 7.** AUROC score corresponding to disability classification according to the EDSS split with threshold 1.5 for the proposed metrics (sorted by AUROC in descending order).

	Metric name	↓AUROC (95% CI)
General	*Response time*	0.64 (95% CI 0.50-0.78)
Demographics	*Age*	0.63 (95% CI 0.53-0.73)
General	*Correct*	0.63 (95% CI 0.49-0.77)
General	*Calibrated rate*	0.59 (95% CI 0.43-0.75)
Fatigability	*Δerrors*	0.55 (95% CI 0.41-0.69)
Fatigability	*Δcorrect*	0.52 (95% CI 0.38-0.66)
General	*Errors*	0.51 (95% CI 0.38-0.64)
Fatigability	*Δresponse time*	0.50 (95% CI 0.36-0.64)

### Differences in predictive power between the best fatigue and disability metrics

**Figure 9.**
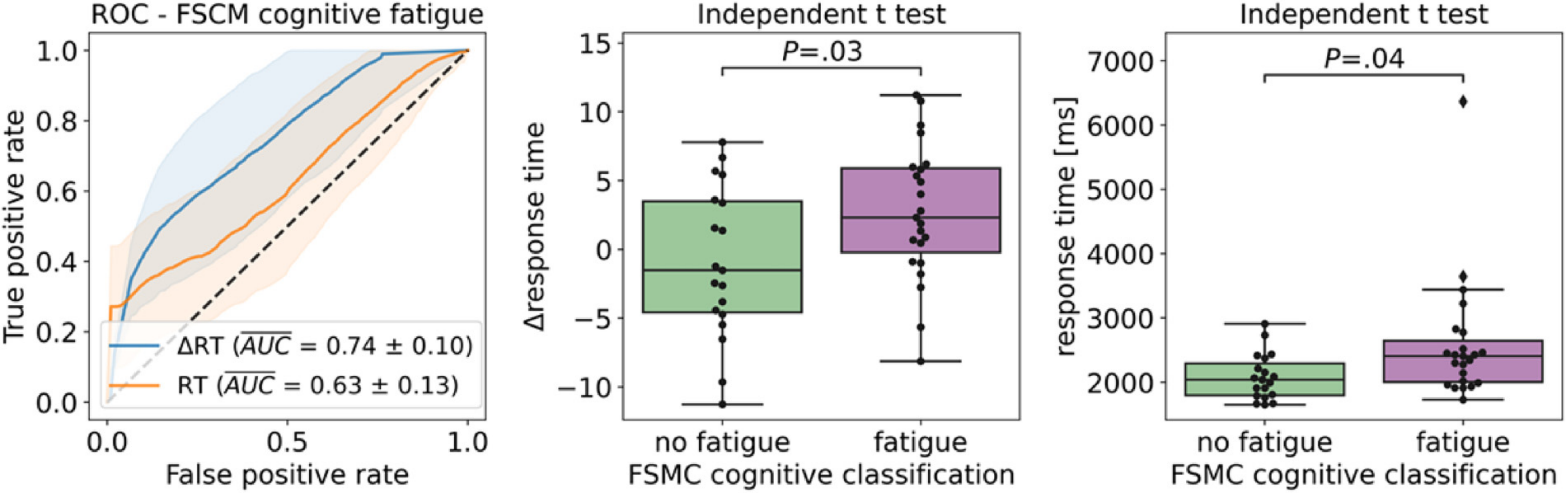
Mean AUROC for cognitive fatigue according to FSMC cognitive subscale (N = 42). ROC curves for Δ*response time* (ΔRT) and *response time* (RT) generated using Monte-Carlo simulation with 1000 iterations (left), t-test results for Δ*response time* (center) and *response time* (right). Δ*response time* and response time show a statistically significant difference between the fatigue groups. *Note.* AUROC, area under the receiver operating characteristic; FSMC, fatigue score for motor and cognitive functions.

 Figure 9 on the left shows a visual representation of the ROC curves corresponding to the FSMC classification for *Δresponse time*, best performing feature to classify cognitive fatigue and *response time*, best performing feature to classify disability. *Δresponse time* outperforms *response time* by 11 percentage points in classifying fatigue according to the FSMC*.* The center of the figure shows boxplots of *Δresponse time* for the groups fatigued and non-fatigued as well as the t-test results. The image displays the statistically significant difference between the fatigue and non-fatigued groups (t = 2.27, *P* = .03). Similarly, the right displays the boxplots corresponding to the *response time*. There is a statistically significant difference between the groups (t = 2.16, *P* = .04). The difference is significant also without the outlier in the fatigue group.

We conducted a one-way analysis of covariance (ANCOVA) to examine whether *response time* differed between fatigue and non-fatigue groups when controlling for EDSS. For this analysis, we did remove the outlier in response time in the fatigue group as the outlier violated the normality assumptions of ANCOVA. We verified the test assumptions: Shapiro-Wilk test indicates the data is normally distributed for the group with no fatigue *W*(19) = .926 (*P* *=* .15) but not for the fatigued group *W*(22) = .899 (*P* = .03). However, as the distribution is close to normal and ANCOVAs are robust to this assumption violation, no steps were taken. Visual analysis with a scatter plot indicates similar regression slopes and an F test indicates no interaction between EDSS and fatigue group *F* *=* (1,37) = .24 (*P* = .64). Finally, Levene's Test confirms the homogeneity of variance with *F(1,39)* *=* 1.27 (*P* = .27). ANCOVA analysis reveals that after controlling for EDSS (disability), there was no significant difference in *response time* between the fatigue groups *F(1,38*) = 1.42, *P* *=* *.*24. For a similar analysis on *correct*, refer to Supplements.

**Figure 10.**
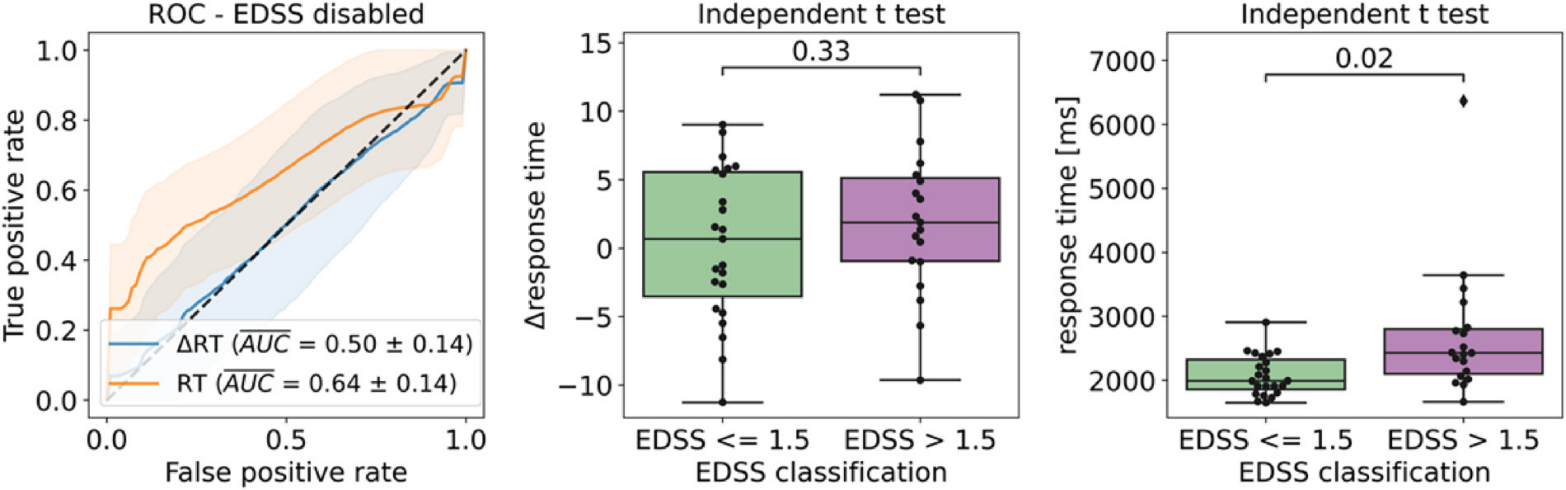
Mean AUROC for disability according to EDSS (N = 42). ROC curves for Δ*response time* (ΔRT) and *response time* (RT) generated using Monte-Carlo simulation with 1000 iterations (left), t-test results for Δ*response time* (center) and *response time* (right). Δ*response time* does not show a statistically significant difference between the disability groups, while *response time* does. *Note.* AUROC, area under the receiver operating characteristic; EDSS, expanded disability status scale; FSMC, fatigue score for motor and cognitive functions.

Figure 10 shows data corresponding to disability classification according to the EDSS threshold. The left side of the Figure 10 shows a visual representation of the ROC curves corresponding to the disability classification for *Δresponse time*, the best performing feature to classify cognitive fatigue and *response time*, the best performing feature to classify disability. In this case, *response time* outperforms *Δresponse time* by 14 percentage points.

## Discussion

We described the development process and pilot study of a new test (cFAST) for cognitive fatigability. Our result provides early evidence that the cFAST measurement could be useful to identify patients with cognitive fatigue, as assessed by the FSMC cognitive subscale. So far, only a few studies assess cognitive fatigability with specific tasks in pwMS.^39,40^ Moreover, previous results are contradictory, with some showing fatigability while others not.^39,40^ Cognitive fatigability studies are in their infancy, and research could benefit from new approaches and validation studies. Our approach differs from previous methods in that it is tailored to patients’ disabilities with its calibration mechanism that also enforces rapid decision-making, which we believe contributes to eliciting cognitive fatigability within a single test session and in a short period. In addition, our smartphone-based test is easy to administer, portable, and designed to be applied outside clinical settings, potentially allowing for remote and frequent monitoring. Concerning cognitive testing, healthy controls and pwMS perceive the PASAT as unpleasant and less likable, while the SDMT is preferred and found appropriate for cognitive testing.^46^ Thus, we believe cFAST will have good acceptance as it follows a similar logic to the SDMT and does not require patients to perform arithmetic operations under pressure like the PASAT. However, user acceptance of the cFAST needs to be assessed in future studies.

### Fatigability metrics relate to fatigue, while general metrics relate to disability

We derived two sets of metrics from cFAST: *fatigability* and *general* metrics. Our initial group-level analysis with a t-test revealed statistically significant differences between fatigued and non-fatigued patients with several *general* and *fatigability* metrics. Overall, we found more significant differences between the groups with the *general* metrics than *fatigability* metrics. However, results from the ANCOVA analysis revealed that EDSS is associated with the metrics *response time* and *correct.* Furthermore*,* the statistical difference in the fatigue groups in terms of these metrics is due to disability and not due to fatigue. Hence, after controlling for EDSS, the statistical difference between the groups disappears. We further analyzed how the groups’ differences related to patients’ disabilities. To this end, we divided our study population into two groups according to EDSS, disabled (EDSS>1.5) and non-disabled (EDSS< = 1.5). This grouping revealed statistically significant differences with the *general* metrics but not with the *fatigability* metrics. This result suggests that *general* metrics are related to and confounded by disability, while this is not true for the *fatigability* metrics. We conducted the AUROC analysis controlling for disability with Monte-Carlo simulations and stratified splits to further rule out the effect of disability from the fatigue analysis. These results confirmed our hypothesis that *fatigability* metrics are better predictors of fatigue than *general* metrics. *Δresponse time*, the best-performing metric to classify fatigue (with an average AUROC of 0.74), is 11 percentage points above *response time*, the best-performing *general* metric for fatigue. Conversely, *general* metrics dominate the disability classification, with *response time* being the best metric (average AUROC of 0.64), 9 percentage points above the best *fatigability* metric *Δerrors*. Analysis of the *fatigability* metrics revealed that, on average, performance during the tests tends to worsen for fatigued patients, while patients without fatigue tend to improve. Previous work on fatigability showed decline towards the end of sustained cognitive activity in pwMS while controls did not.^20,44^ Our findings go in line with these results. However, our analysis focused only on pwMS to decrease disease-specific confoundings.

### Consideration for remote and unsupervised monitoring

We designed and implemented cFAST to achieve remote monitoring. Hence, cFAST seeks to be self-explanatory. For instance, trials aim at familiarizing the users with the core test logic of matching numbers to symbols following the shown mapping rule. Thanks to the feedback displayed after every answer, users can quickly realize when they are making mistakes. The immediate feedback, together with the requirement of at least 70% correct answers out of a minimum of 20, helps us determine if the user has correctly understood the test logic and the requirement to perform it quickly. As described in the methods section, we derived the pace of the cFAST, *calibrated rate*, from the calibration phase. The speed requirement seeks to induce cognitive fatigability in a short period. *Calibrated rate* is derived for each patient, personalizing the test and adjusting for the different disability spectrum and baseline performance of the patients.

### Implications of objective measurement of cognitive fatigue

A reliable and objective measurement of fatigability would help quantify the effectiveness of treatments, both in clinical trials and routine care, and it would also help clinicians distinguish between confounding comorbidities. Several randomized placebo-controlled clinical trials tested different compounds for treating fatigue.^11–18,67,68^ However, results from these clinical trials are inconsistent. A common denominator in these trials is that they quantified the outcome measure using subjective questionnaires. It is known that the magnitude of the placebo effect is an important reason for the variability in the efficacy during trials.^16,69,70^ Thus, an objective measurement would help clinicians overcome these limitations and complement questionnaires to evaluate treatments’ efficacy. Our evaluation shows that cFAST is a promising tool for quantifying cognitive fatigue. Additionally, we believe the design of cFAST will allow remote and unsupervised monitoring, enabling more frequent assessments and detailed fatigue profiles of patients while reducing the cost associated with medical personnel and specialized equipment.

### Limitations and future work

A limitation of our study is the lack of a gold standard cognitive fatigability assessment to validate our approach. Currently, there is no established method to quantify cognitive fatigability. Up until now, existing research has used cognitive tests protracted for extended periods as an attempt to induce and quantify fatigability. However, these approaches tend to be long, tedious, and costly. Moreover, results from these experiments are inconclusive. Hence, we directly compared our metrics to a widely accepted and validated fatigue questionnaire within MS research, the FSMC. The FSMC has the advantage of offering a subscale to evaluate cognitive fatigue independently of physical fatigue. Another limitation of our study is our sample size, limited to 42 study participants. We are aware that more extensive evaluations are needed to determine if the test can be established as a surrogate for perceived cognitive fatigue for clinical decision-making. In particular, our pilot study uses a cross-sectional design, thus, we are not able to define the clinical significance in the changes on the fatigability scores in individual patients. Future studies are needed to address this question. Finally, we designed cFAST to be suitable for remote and unsupervised monitoring. However, in this study, the evaluation was conducted within the hospital in a controlled environment. Further studies including longitudinal outside-the-hospital evaluations in larger MS cohorts and within-subjects comparison are needed to confirm the results. Nevertheless, we believe our study offers a detailed evaluation of our newly developed cognitive fatigability test.

As part of future work and prior to the clinical implementation more data has to be generated to further evaluate the generalization of the adaptation phase. Additionally, our study highlights the need for implementing changes to improve the data quality in an unsupervised setting. First, we recommend incorporating a statement in the cFAST instructions about the importance of conducting the test in a distraction-free environment (i.e. activate “do not disturb” modality, use quiet room). Second, we recommend automatically dismissing test sessions if no input is recorded within a certain period after the start. Distractions in uncontrolled environments (e.g. incoming phone calls or messages) can result in empty test sessions or significant periods without data, thus producing erroneous values for the proposed set of metrics. Moreover, future studies should examine whether cFAST could aid clinicians distinguishing between confounding such as depression, sleepiness, or others. Finally, we need to investigate further the frequency that patients need to conduct the calibration phase in unsupervised settings. However, we believe that calibration has to be performed only once and that the calibrated rate can be recomputed, if necessary, directly from the existing patients’ cFAST sessions. Nonetheless, this requires further studies, including longitudinal data.

## Conclusions

We introduced cFAST, a novel smartphone-based test to quantify cognitive fatigability tailored to the user's disability by its calibration mechanism. With cFAST, we aim at having an objective surrogate of fatigue that allows monitoring of individual patients over time in uncontrolled environments (e.g. at home). We do not aim to have a diagnostic tool, but rather a solution for clinicians to make informed and timely decisions as to whether a patient's condition is improving or deteriorating and act accordingly. Results from our pilot study provide evidence supporting the validity of our approach and show that the *fatigability* metrics could potentially be used as a surrogate for perceived cognitive fatigue and motivate further research in this area.

## Supplemental Material

sj-docx-1-dhj-10.1177_20552076221117740 - Supplemental material for Cognitive fatigability assessment test (cFAST): Development of a new instrument to assess cognitive fatigability and pilot study on its association to perceived fatigue in multiple sclerosis Click here for additional data file. Supplemental material, sj-docx-1-dhj-10.1177_20552076221117740 for Cognitive fatigability assessment test (cFAST): Development of a new instrument to assess cognitive fatigability and pilot study on its association to perceived fatigue in multiple sclerosis by Liliana Barrios, Rok Amon, Pietro Oldrati, Marc Hilty, Christian Holz and Andreas Lutterotti in Digital Health

## References

[1] Friedman JH Brown RG Comella C , et al. Fatigue in Parkinson’s disease: a review. Mov Disord 2007; 22: 297–308. 17133511 10.1002/mds.21240

[2] Krupp LB Alvarez LA LaRocca NG , et al. Fatigue in multiple sclerosis. Arch Neurol 1988; 45: 435–437. 3355400 10.1001/archneur.1988.00520280085020

[3] Mandal S Barnett J Brill SE , et al. ‘Long-COVID’: a cross-sectional study of persisting symptoms, biomarker and imaging abnormalities following hospitalisation for COVID-19. Thorax 2021; 76: 396–398. 33172844 10.1136/thoraxjnl-2020-215818 PMC7615158

[4] Induruwa I Constantinescu CS Gran B . Fatigue in multiple sclerosis - a brief review. J Neurol Sci 2012; 323: 9–15. 22935407 10.1016/j.jns.2012.08.007

[5] Kobelt G Thompson A Berg J , et al. New insights into the burden and costs of multiple sclerosis in Europe. Mult Scler 2017; 23: 1123–1136. 28273775 10.1177/1352458517694432 PMC5476197

[6] Krupp LB McLinskey N MacAllister WS . Fatigue in multiple sclerosis. In: Multiple Sclerosis Therapeutics. 3rd ed. CRC Press, 2007, pp. 805–818.

[7] Valko PO Bassetti CL Bloch KE , et al. Validation of the fatigue severity scale in a Swiss cohort. Sleep 2008; 31: 1601–1607. 19014080 10.1093/sleep/31.11.1601 PMC2579971

[8] Téllez N Río J Tintoré M , et al. Does the modified fatigue impact scale offer a more comprehensive assessment of fatigue in MS? Mult Scler 2005; 11: 198–202. 15794395 10.1191/1352458505ms1148oa

[9] Penner IK Raselli C Stöcklin M , et al. The fatigue scale for motor and cognitive functions (FSMC): validation of a new instrument to assess multiple sclerosis-related fatigue. Mult Scler 2009; 15: 1509–1517. 19995840 10.1177/1352458509348519

[10] Penner I-K Paul F . Fatigue as a symptom or comorbidity of neurological diseases. Nat Rev Neurol 2017; 13: 662–675. 29027539 10.1038/nrneurol.2017.117

[11] Brioschi A Gramigna S Werth E , et al. Effect of modafinil on subjective fatigue in multiple sclerosis and stroke patients. Eur Neurol 2009; 62: 243–249. 19672078 10.1159/000232927

[12] The Canadian MS Research Group. A randomized controlled trial of amantadine in fatigue associated with multiple sclerosis. Can J Neurol Sci 1987; 14: 273–278. 2889518 10.1017/s0317167100026603

[13] Krupp LB Coyle PK Doscher C , et al. Fatigue therapy in multiple sclerosis: results of a double-blind, randomized, parallel trial of amantadine, pemoline, and placebo. Neurology 1995; 45: 1956–1961. 7501140 10.1212/wnl.45.11.1956

[14] Lange R Volkmer M Heesen C , et al. Modafinil effects in multiple sclerosis patients with fatigue. J Neurol 2009; 256: 645–650. 19367356 10.1007/s00415-009-0152-7

[15] Möller F Poettgen J Broemel F , et al. HAGIL (Hamburg Vigil Study): a randomized placebo-controlled double-blind study with modafinil for treatment of fatigue in patients with multiple sclerosis. Mult Scler 2011; 17: 1002–1009. 21561959 10.1177/1352458511402410

[16] Nourbakhsh B Revirajan N Morris B , et al. Safety and efficacy of amantadine, modafinil, and methylphenidate for fatigue in multiple sclerosis: a randomised, placebo-controlled, crossover, double-blind trial. The Lancet Neurology 2021; 20: 38–48. 33242419 10.1016/S1474-4422(20)30354-9 PMC7772747

[17] Rammohan KW Rosenberg JH Lynn DJ , et al. Efficacy and safety of modafinil (Provigil) for the treatment of fatigue in multiple sclerosis: a two centre phase 2 study. J Neurol Neurosurg Psychiatry 2002; 72: 179–183. 11796766 10.1136/jnnp.72.2.179 PMC1737733

[18] Stankoff B Waubant E Confavreux C , et al. Modafinil for fatigue in MS: a randomized placebo-controlled double-blind study. Neurology 2005; 64: 1139–1143. 15824337 10.1212/01.WNL.0000158272.27070.6A

[19] Kluger BM Krupp LB Enoka RM . Fatigue and fatigability in neurologic illnesses: proposal for a unified taxonomy. Neurology 2013; 80: 409–416. 23339207 10.1212/WNL.0b013e31827f07be PMC3589241

[20] Schwid SR Tyler CM Scheid EA , et al. Cognitive fatigue during a test requiring sustained attention: a pilot study. Mult Scler 2003; 9: 503–508. 14582777 10.1191/1352458503ms946oa

[21] Krupp LB Elkins LE . Fatigue and declines in cognitive functioning in multiple sclerosis. Neurology 2000; 55: 934–939. 11061247 10.1212/wnl.55.7.934

[22] Walker LAS Lindsay-Brown AP Berard JA . Cognitive fatigability interventions in neurological conditions: a systematic review. Neurol Ther 2019; 8: 251–271. 31586303 10.1007/s40120-019-00158-3 PMC6858900

[23] Dobkin BH . Fatigue versus activity-dependent fatigability in patients with central or peripheral motor impairments. Neurorehabil Neural Repair 2008; 22: 105–110. 18285599 10.1177/1545968308315046 PMC4160309

[24] Steens A de Vries A Hemmen J , et al. Fatigue perceived by multiple sclerosis patients is associated with muscle fatigue. Neurorehabil Neural Repair 2012; 26: 48–57. 21856990 10.1177/1545968311416991

[25] Loy BD Taylor RL Fling BW , et al. Relationship between perceived fatigue and performance fatigability in people with multiple sclerosis: a systematic review and meta-analysis. J Psychosom Res 2017; 100: 1–7. 28789787 10.1016/j.jpsychores.2017.06.017 PMC5875709

[26] Wolkorte R Heersema DJ Zijdewind I . Muscle fatigability during a sustained Index finger abduction and depression scores are associated with perceived fatigue in patients with relapsing-remitting multiple sclerosis. Neurorehabil Neural Repair 2015; 29: 796–802. 25605633 10.1177/1545968314567151

[27] Barrios L Oldrati P Hilty M , et al. Smartphone-based tapping frequency as a surrogate for perceived fatigue: an in-the-wild feasibility study in multiple sclerosis patients. Proc ACM Interact Mob Wearable Ubiquitous Technol 2021; 5: 1–30.

[28] Walker LAS Berard JA Berrigan LI , et al. Detecting cognitive fatigue in multiple sclerosis: method matters. J Neurol Sci 2012; 316: 86–92. 22336698 10.1016/j.jns.2012.01.021

[29] Morrow SA Rosehart H Johnson AM . Diagnosis and quantification of cognitive fatigue in multiple sclerosis. Cogn Behav Neurol 2015; 28: 27–32. 25812128 10.1097/WNN.0000000000000050

[30] Berard JA Smith AM Walker LAS . A longitudinal evaluation of cognitive fatigue on a task of sustained attention in early relapsing-remitting multiple sclerosis. Int J MS Care 2018; 20: 55–61. 29670491 10.7224/1537-2073.2016-106 PMC5898916

[31] Berard JA Fang Z Walker LAS , et al. Imaging cognitive fatigability in multiple sclerosis: objective quantification of cerebral blood flow during a task of sustained attention using ASL perfusion fMRI. Brain Imaging Behav 2020; 14: 2417–2428. 31468375 10.1007/s11682-019-00192-7

[32] van der Linden D Frese M Meijman TF . Mental fatigue and the control of cognitive processes: effects on perseveration and planning. Acta Psychol 2003; 113: 45–65. 10.1016/s0001-6918(02)00150-6 12679043

[33] Möller MC de Boussard C N Oldenburg C , et al. An investigation of attention, executive, and psychomotor aspects of cognitive fatigability. J Clin Exp Neuropsychol 2014; 36: 716–729. 24965830 10.1080/13803395.2014.933779

[34] Wang C Ding M Kluger BM . Change in intraindividual variability over time as a key metric for defining performance-based cognitive fatigability. Brain Cogn 2014; 85: 251–258. 24486386 10.1016/j.bandc.2014.01.004 PMC3980793

[35] Burke SE Babu Henry Samuel I Zhao Q , et al. Task-based cognitive fatigability for older adults and validation of mental fatigability subscore of Pittsburgh Fatigability Scale. Front Aging Neurosci 2018; 10: 327. 30405396 10.3389/fnagi.2018.00327 PMC6202947

[36] Tombaugh TN . A comprehensive review of the Paced Auditory Serial Addition Test (PASAT). Arch Clin Neuropsychol 2006; 21: 53–76. 16290063 10.1016/j.acn.2005.07.006

[37] Basner M Dinges DF . Maximizing sensitivity of the psychomotor vigilance test (PVT) to sleep loss. Sleep 2011; 34: 581–591. 21532951 10.1093/sleep/34.5.581 PMC3079937

[38] Stroop JR . Studies of interference in serial verbal reactions. J Exp Psychol 1935; 18: 643–662.

[39] DeLuca J Genova HM Hillary FG , et al. Neural correlates of cognitive fatigue in multiple sclerosis using functional MRI. J Neurol Sci 2008; 270: 28–39. 18336838 10.1016/j.jns.2008.01.018

[40] Chen MH Wylie GR Sandroff BM , et al. Neural mechanisms underlying state mental fatigue in multiple sclerosis: a pilot study. J Neurol 2020; 267: 2372–2382. 32350648 10.1007/s00415-020-09853-w

[41] Pattyn N Neyt X Henderickx D , et al. Psychophysiological investigation of vigilance decrement: boredom or cognitive fatigue? Physiol Behav 2008; 93: 369–378. 17999934 10.1016/j.physbeh.2007.09.016

[42] Agyemang C Berard JA Walker LAS . Cognitive fatigability in multiple sclerosis: how does performance decline over time on the paced auditory serial addition test? Mult Scler Relat Disord 2021; 54: 103130. 34273611 10.1016/j.msard.2021.103130

[43] Berard JA Walker LAS . Increasing the clinical utility of the paced auditory serial addition test: normative data for standard, dyad, and cognitive fatigability scoring. Cogn Behav Neurol 2021; 34: 107. 34074865 10.1097/WNN.0000000000000268

[44] Bryant D Chiaravalloti ND DeLuca J . Objective measurement of cognitive fatigue in multiple sclerosis. Rehabil Psychol 2004; 49: 114–122.

[45] Fisk JD Archibald CJ . Limitations of the paced auditory serial addition test as a measure of working memory in patients with multiple sclerosis. J Int Neuropsychol Soc 2001; 7: 363–372. 11311037 10.1017/s1355617701733103

[46] Walker LAS Cheng A Berard J , et al. Tests of information processing speed: what do people with multiple sclerosis think about them? Int J MS Care 2012; 14: 92–99. 24453739 10.7224/1537-2073-14.2.92 PMC3883004

[47] Smith A . Symbol digit modalities test (SDMT) manual (revised) Western psychological services. *Los Angeles*.

[48] Benedict RHB Smerbeck A Parikh R , et al. Reliability and equivalence of alternate forms for the Symbol Digit Modalities Test: implications for multiple sclerosis clinical trials. Mult Scler 2012; 18: 1320–1325. 22277740 10.1177/1352458511435717

[49] Roar M Illes Z Sejbaek T . Practice effect in Symbol Digit Modalities Test in multiple sclerosis patients treated with natalizumab. Mult Scler Relat Disord 2016; 10: 116–122. 27919477 10.1016/j.msard.2016.09.009

[50] Patel VP Walker LAS Feinstein A . Deconstructing the symbol digit modalities test in multiple sclerosis: the role of memory. Mult Scler Relat Disord 2017; 17: 184–189. 29055455 10.1016/j.msard.2017.08.006

[51] van Oirschot P Heerings M Wendrich K , et al. Symbol digit modalities test variant in a smartphone app for persons with multiple sclerosis: validation study. JMIR Mhealth Uhealth 2020; 8: e18160. 33016886 10.2196/18160 PMC7573704

[52] Griffin N Kehoe M . A questionnaire study to explore the views of people with multiple sclerosis of using smartphone technology for health care purposes. Disabil Rehabil 2018; 40: 1434–1442. 28322588 10.1080/09638288.2017.1300332

[53] Apolinário-Hagen J Menzel M Hennemann S , et al. Acceptance of Mobile health apps for disease management among people with multiple sclerosis: web-based survey study. JMIR Form Res 2018; 2: e11977. 30684408 10.2196/11977 PMC6334710

[54] Ayobi A Marshall P Cox AL , et al. Quantifying the body and caring for the mind: self-tracking in multiple sclerosis. In: Proceedings of the 2017 CHI conference on human factors in computing systems. New York, NY, USA: Association for Computing Machinery, 2017, pp.6889–6901.

[55] Giunti G Kool J Rivera Romero O , et al. Exploring the specific needs of persons with multiple sclerosis for mHealth solutions for physical activity: mixed-methods study. JMIR Mhealth Uhealth 2018; 6: e37. 29426814 10.2196/mhealth.8996 PMC5889817

[56] Van Kessel K Babbage DR Reay N , et al. Mobile technology use by people experiencing multiple sclerosis fatigue: survey methodology. JMIR Mhealth Uhealth 2017; 5: e6. 28246073 10.2196/mhealth.6192 PMC5350455

[57] Midaglia L Mulero P Montalban X , et al. Adherence and satisfaction of smartphone- and smartwatch-based remote active testing and passive monitoring in people with multiple sclerosis: nonrandomized interventional feasibility study. J Med Internet Res 2019; 21: e14863. 31471961 10.2196/14863 PMC6743265

[58] Rice DR Kaplan TB Hotan GC , et al. Electronic pill bottles to monitor and promote medication adherence for people with multiple sclerosis: a randomized, virtual clinical trial. J Neurol Sci 2021; 428: 117612. 34392138 10.1016/j.jns.2021.117612

[59] Motl RW Hubbard EA Bollaert RE , et al. Randomized controlled trial of an e-learning designed behavioral intervention for increasing physical activity behavior in multiple sclerosis. Mult Scler J Exp Transl Clin 2017; 3: 2055217317734886. 29051831 10.1177/2055217317734886 PMC5637983

[60] Zhai S Kong J Ren X . Speed--accuracy tradeoff in Fitts’ law tasks—on the equivalency of actual and nominal pointing precision. Int J Hum Comput Stud 2004; 61: 823–856.

[61] World Medical Association. World Medical Association Declaration of Helsinki: ethical principles for medical research involving human subjects. JAMA 2013; 310: 2191–2194. 24141714 10.1001/jama.2013.281053

[62] Möckel T Beste C Wascher E . The effects of time on task in response selection--an ERP study of mental fatigue. Sci Rep 2015; 5: 10113. 26054837 10.1038/srep10113 PMC4460573

[63] Wascher E Rasch B Sänger J , et al. Frontal theta activity reflects distinct aspects of mental fatigue. Biol Psychol 2014; 96: 57–65. 24309160 10.1016/j.biopsycho.2013.11.010

[64] Schwid SR Thornton CA Pandya S , et al. Quantitative assessment of motor fatigue and strength in MS. Neurology 1999; 53: 743–750. 10489035 10.1212/wnl.53.4.743

[65] Barrios L Oldrati P Lindlbauer D , et al. A rapid tapping task on commodity smartphones to assess motor fatigability. In: Proceedings of the 2020 CHI conference on human factors in computing systems. New York, NY, USA: Association for Computing Machinery, 2020, pp.1–10.

[66] Preacher KJ Selig JP . Advantages of Monte Carlo confidence intervals for indirect effects. Commun Methods Meas 2012; 6: 77–98.

[67] Cohen RA Fisher M . Amantadine treatment of fatigue associated with multiple sclerosis. Arch Neurol 1989; 46: 676–680. 2730380 10.1001/archneur.1989.00520420096030

[68] Ledinek AH Sajko MC Rot U . Evaluating the effects of amantadin, modafinil and acetyl-L-carnitine on fatigue in multiple sclerosis--result of a pilot randomized, blind study. Clin Neurol Neurosurg 2013; 115: S86–S89. 24321164 10.1016/j.clineuro.2013.09.029

[69] Pucci E Branãs P D’Amico R , et al. Amantadine for fatigue in multiple sclerosis. Cochrane Database Syst Rev 2007; 2007(1). DOI: 10.1002/14651858. PMC6991937 17253480

[70] Sheng P Hou L Wang X , et al. Efficacy of modafinil on fatigue and excessive daytime sleepiness associated with neurological disorders: a systematic review and meta-analysis. PLoS One 2013; 8: e81802. 24312590 10.1371/journal.pone.0081802 PMC3849275

