# Supplementary material for: Cognitive fatigability assessment test (cFAST): Development of a new instrument to assess cognitive fatigability and pilot study on its association to perceived fatigue in multiple sclerosis
Source: Digit Health. 2022 Aug 25;8:20552076221117740. doi: 10.1177/20552076221117740 (PMC9421030; doi:10.1177/20552076221117740)
Supplement: sj-docx-1-dhj-10.1177_20552076221117740 - Supplemental material for Cognitive fatigability assessment test (cFAST): Development of a new instrument to assess cognitive fatigability and pilot study on its association to perceived fatigue in multiple sclerosis [file sj-docx-1-dhj-10.1177_20552076221117740.docx]

## Supplements

### User interface designs and selection

We designed five user interfaces as prospects for our cognitive fatigability test, these are depicted in Figure S1. We created two test modaties: single or grid modality. In the single modality, users have to map the middle symbol to its corresponding key as displayed in the top mapping rule located on the top of the screen. While on the grid modality, users are presented with a four by three grid composed of six symbols and six keys. During each round, users have to map the elements within the grip following the mapping rule presented on top. The grid modality designs (Figure S1 bottom row) were discarded after discussion with the neurologists and neuropsychologists. One of the main arguments against the grid design was that the added complexity would also result in more difficulties for making a fair comparison between the patients. The single modality design was further discussed with our specialist team and also shown to a group of patients attending the in-patient clinic. We presented a printed version as well as a digital version of each of the three single modality tests and asked for the preference in terms of style. After discussion with the specialist and informal feedback from the patients on the design we opted for the SDMT style symbols as the other symbols were too similar between each other making the selection harder and being more error prone due to confusion.


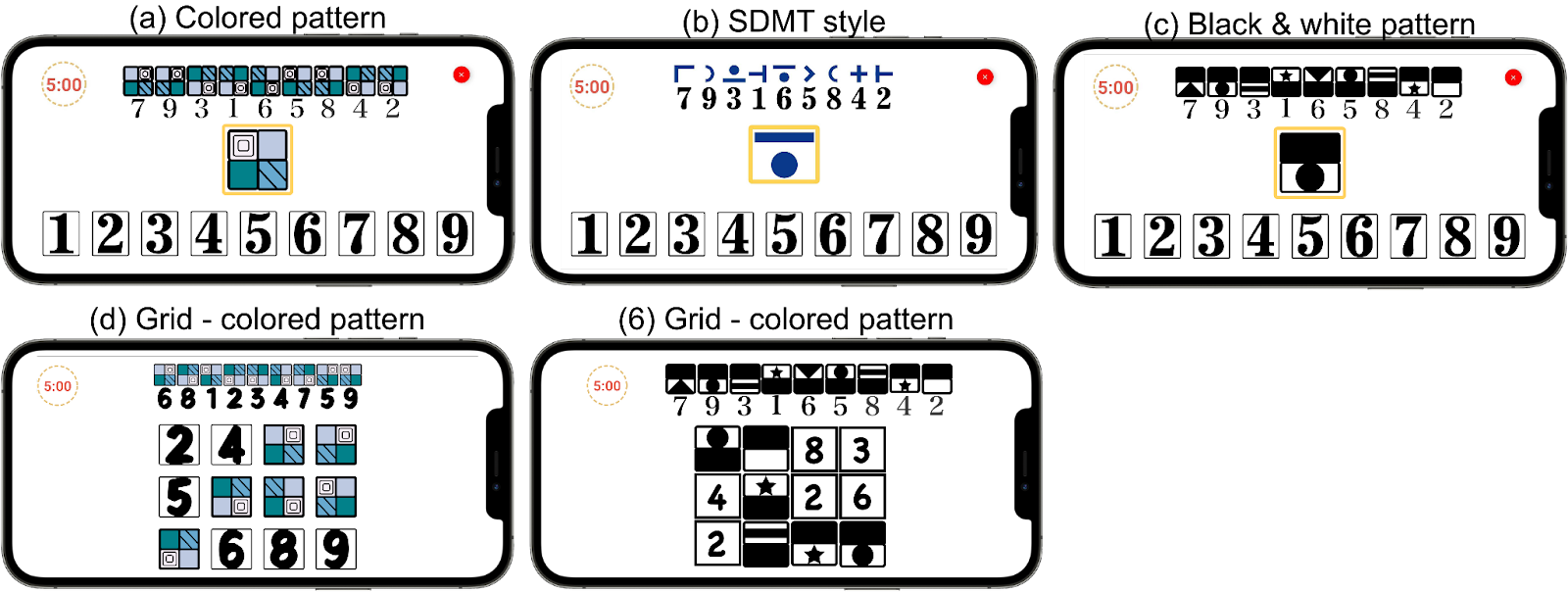


**Figure S1:** Different user interfaces considered for cFAST. The top row depicts tests with a single selection option. The bottom row depicts a mapping grid, where users need to map each symbol within the grid with its corresponding key as displayed in the top mapping rule.

**Table S1:** Demographic characteristics of participants grouped by disability (split with threshold 1.5).

|  |  | not disabled | disabled | *P* |
| --- | --- | --- | --- | --- |
| number | | 23 | 19 |  |
| age, mean (SD) | | 33.70 (8.93) | 42.37 (13.77) | .03 |
| **gender, n (%)** | |  |  |  |
|  | m | 6 (26) | 8 (42) | .44 |
|  | w | 17 (74) | 11 (58) |  |
| **MS type, n (%)** | |  |  |  |
|  | PMS | 0 (5) | 4 (21) | .04 |
|  | RRMS | 23 (100) | 15 (79) |  |
| **Disease duration, mean (SD)** | | 9.61 (5.98) | 13.16 (8.77) | 0.14 |
| **DMT, n (%)** | |  |  |  |
|  | None | 2 (9) | 0 (0) |  |
|  | Interferon beta-1a | 1 (4) | 0 (0) |  |
|  | Dimethyl fumarate | 3 (13) | 0 (0) |  |
|  | Teriflunomide | 1 (4) | 1 (5) |  |
|  | Glatiramer acetate | 1 (4) | 1 (5) |  |
|  | Fingolimod | 2 (9) | 0 (0) |  |
|  | Natalizumab | 7 (30) | 7 (37) |  |
|  | Rituximab | 0 (0) | 4 (21) |  |
|  | Ocrelizumab | 6 (26) | 6 (32) |  |
| **Fatigue medication, n (%)** | |  |  |  |
|  | None | 23 (100) | 18 (95) | 1.00 |
|  | Modafinil | 0 (0) | 1 (5) |  |
| EDSS, mean (SD) | | 0.54 (0.67) | 3.26 (1.54) | <.001 |
| **FSMC, mean (SD)** | |  |  |  |
|  | total | 39.78 (18.26) | 60.53 (19.48) | .001 |
|  | cognitive | 19.13 (9.55) | 29.47 (10.39) | .002 |
|  | motor | 20.65 (9.22) | 31.05 (10.28) | .002 |

Data are mean (SD) or n (%). PMS: progressive multiple sclerosis; RRMS: relapsing-remitting multiple sclerosis; Disease duration is measured in years since first manifestation; EDSS: expanded disability status scale; FSMC: Fatigue Score for motor functions and cognition; DMT: disease modifying therapy.

**Table S2:** Metrics comparison between fatigued and non fatigued patients with mean (SD), standard deviation, and Mann-Whitney U test (two-tailed) to assess whether there is a statistically significant difference between the groups.

|  |  | no fatigue (n=19) | cognitive fatigue (n=23) | U | *P* |
| --- | --- | --- | --- | --- | --- |
| *response time* |  | 2083.3 (358.31) | 2586.88 (961.28) | 316.0 | .01 |
| *calibrated rate* |  | 3289.47 (1229.75) | 3922.91 (1396.06) | 298.0 | .045 |
| *correct* |  | 109.11 (15.97) | 90.96 (24.21) | 119.5 | .01 |
| *errors* |  | 7.58 (6.07) | 8.04 (4.13) | 243.5 | .53 |
| *Δcorrect* |  | 3.51 (11.19) | -2.73 (9.95) | 147.0 | .07 |
| *Δresponse time* |  | -0.96 (5.5) | 2.69 (4.94) | 298.0 | .045 |
| *Δerrors* |  | -0.46 (2.05) | 0.03 (1.86) | 255.5 | .35 |

**Table S3:** Metrics comparison between disabled and not disabled patients with mean (SD), standard deviation, and Mann-Whitney U test (two-tailed) to assess whether there is a statistically significant difference between the groups.

|  |  | not disabled (n=23) | disabled (n=19) | U | *P* |
| --- | --- | --- | --- | --- | --- |
| *response time* |  | 2080.23 (317.39) | 2696.61 (1030.37) | 332.0 | .004 |
| *calibrated rate* |  | 3211.22 (840.63) | 4151.0 (1658.95) | 312.0 | .02 |
| *correct* |  | 108.3 (15.1) | 88.11 (25.44) | 105.5 | .004 |
| *errors* |  | 7.96 (5.69) | 7.68 (4.26) | 220.0 | .97 |
| *Δcorrect* |  | 0.55 (10.68) | -0.47 (11.35) | 208 | ​​.80 |
| *Δresponse time* |  | 0.29 (5.55) | 1.95 (5.33) | 252.0 | .40 |
| *Δerrors* |  | -0.03 (2.05) | -0.4 (1.83) | 198.5 | .61 |

### ANCOVA analysis

We conducted a one-way analysis of covariance (ANCOVA) to examine whether *correct* differed between the fatigue and non-fatigue groups when controlling for EDSS. We verified the test assumptions: Shapiro-Wilk test indicates the data is normally distributed for the group with no fatigue *W*(19)=.96 (*P=*.55) and for the fatigued group *W*(23)=.92 (*P*=.06). Visual analysis with a scatter plot indicates similar regression slopes and an F test indicates no interaction between EDSS and fatigue groups (homogeneity of regression slopes) *F=*(1,38)=.07 (*P*=.8). Finally, Levene’s Test confirms the homogeneity of variance with *F(1,40)=*1.36 (*P*=.25). ANCOVA analysis reveals that after controlling for EDSS (disability), there was no significant difference in fatigue on the *correct* score, *F(1,39*)=2.36 (*P=.*13). Estimated marginal means for no fatigue (M=104.48, SE=4.50) and fatigued (M=94.775, SE=4.06). EDSS is significantly related to *correct* (*F(1,39)=*11.07 *P=*.002). However, if we do not consider EDSS when analyzing fatigue then there is a difference in terms of *correct* between the groups F(1,40)=7.84, *P=.008.*
